# (*Z*)-*N*′-Hy­droxy-4-(trifluoro­meth­yl)benzimidamide

**DOI:** 10.1107/S1600536811005022

**Published:** 2011-03-12

**Authors:** Fei Liu, Fang Zhang, Qifan Chen, Mingdong Dong

**Affiliations:** a College of Chemical Engineering & Materials, Eastern Liaoning University, No. 325 Wenhua Road, Yuanbao District, Dandong City, Liaoning Province 118003, People’s Republic of China; b Experiment Center, Eastern Liaoning University, No. 325 Wenhua Road, Yuanbao District, Dandong City, Liaoning Province 118003, People’s Republic of China

## Abstract

In the title compound, C_8_H_7_F_3_N_2_O, the OH and NH_2_ substituents adopt a *Z* configuration with respect to the C=N bond. The hy­droxy­imidamide unit is almost planar (r.m.s. deviation = 0.007 Å) and subtends an angle of 26.25 (13)° with the benzene ring. The F atoms of the trifluoro­methyl substituent are disordered over two sets of sites with an occupancy ratio of 0.783 (15):0.217 (15). In the crystal, O—H⋯N hydrogen bonds form centrosymmetric dimers. Additional N—H⋯O hydrogen bonds link the dimers into zigzag chains along the *b* axis. Weak inter­molecular F⋯F contacts of 2.714 (5) Å are also observed.

## Related literature

For the preparation of the title compound, see: Rai *et al.* (2010 ▶). For the use of oxime derivatives in crystal engineering, see: Aakeröy *et al.* (2000 ▶). For a related structure, see: Orama & Saarinen (1996 ▶).
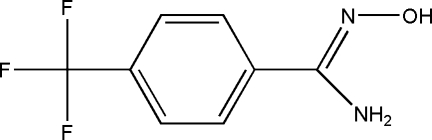


## Experimental

### 

#### Crystal data

C_8_H_7_F_3_N_2_O
*M* *_r_* = 204.16
Monoclinic, 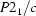

*a* = 9.8706 (8) Å
*b* = 11.2540 (12) Å
*c* = 8.4033 (7) Å
β = 104.61 (2)°
*V* = 903.29 (16) Å^3^
*Z* = 4
Mo *K*α radiation
μ = 0.14 mm^−1^
*T* = 293 K
0.32 × 0.24 × 0.20 mm

#### Data collection

Rigaku R-AXIS RAPID diffractometer
Absorption correction: multi-scan (*ABSCOR*; Higashi, 1995 ▶) *T*_min_ = 0.946, *T*_max_ = 0.972
8605 measured reflections
2058 independent reflections
1324 reflections with *I* > 2σ(*I*)
*R*_int_ = 0.077

#### Refinement

*R*[*F* ^2^ > 2σ(*F* ^2^)] = 0.056
*wR*(*F* ^2^) = 0.183
*S* = 1.07
2058 reflections
164 parameters
42 restraints
H atoms treated by a mixture of independent and constrained refinement
Δρ_max_ = 0.23 e Å^−3^
Δρ_min_ = −0.29 e Å^−3^

### 

Data collection: *PROCESS-AUTO* (Rigaku, 2006 ▶); cell refinement: *PROCESS-AUTO*; data reduction: *CrystalStructure* (Rigaku, 2007 ▶); program(s) used to solve structure: *SHELXS97* (Sheldrick, 2008 ▶); program(s) used to refine structure: *SHELXL97* (Sheldrick, 2008 ▶); molecular graphics: *ORTEP-3 for Windows* (Farrugia, 1997 ▶); software used to prepare material for publication: *WinGX* (Farrugia, 1999 ▶).

## Supplementary Material

Crystal structure: contains datablocks global, I. DOI: 10.1107/S1600536811005022/sj5105sup1.cif

Structure factors: contains datablocks I. DOI: 10.1107/S1600536811005022/sj5105Isup2.hkl

Additional supplementary materials:  crystallographic information; 3D view; checkCIF report

## Figures and Tables

**Table 1:** Hydrogen-bond geometry (Å, °)

*D*—H⋯*A*	*D*—H	H⋯*A*	*D*⋯*A*	*D*—H⋯*A*
N1—H1*N*⋯O1^i^	0.84 (3)	2.36 (3)	3.165 (3)	161 (3)
O1—H1*O*⋯N2^ii^	0.86 (3)	1.98 (3)	2.766 (2)	152 (3)

Symmetry codes: (i) 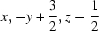; (ii) 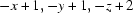.

**Fig. 1.**
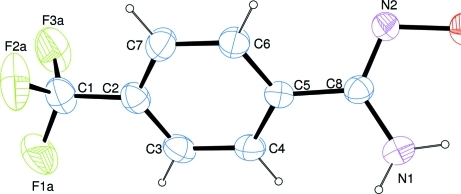
Structure of the title compound with 50% probability displacement ellipsoids. For clarity, only one of the two equivalent disorder components is shown.

**Fig. 2.**
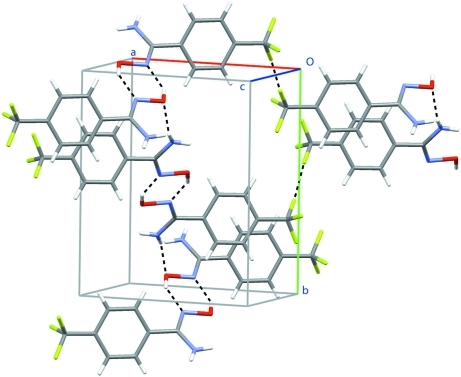
Crystal packing of the title compound viewed down the c axis. Hydrogen bonds and F···F contacts are drawn as dashed lines.

## supplementary crystallographic information

### Comment

The oxime functionality is well known in organic synthesis, analytical chemistry, and coordination chemistry, yet it has remained relatively unexplored as an intermolecular connector in crystal engineering (Aakeröy *et al.*, 2000).

In the title compound, the oxime also carries an amine substituent and assumes a *Z* configuration with respect to the C8═N2 bond (Fig. 1). Atoms F1A:F3B, F2A:F1B, F3A: F2B are disordered over two positions and with site occupancies of 0.5:0.5. The C8,N1,N2,O1 hydroxyimidamide unit is almost planar (r.m.s. deviation 0.007 Å) and subtends an angle of 26.25 (13)° to the C2···C7 benzene ring. The torsion angle O1—N2—C8—C5 between the oxime unit and the ring system is -177.71 (15)°. In the crystal O1–H1O···N1 hydrogen bonds form centrosymmetric dimers. An additional N1–H1N···O1 hydrogen bond links these dimers into zigzag chains along *b*. Weak intermolecular F2A···F2A^iii^ contacts, 2.714 (5) Å, (^iii^ = -*x*, 1-*y*, -*z*) are also observed (Fig. 2).

### Experimental

The compound was prepared by a reported procedure  (Rai *et al.*, 2010) To a solution of 4-(trifluoromethyl)benzonitrile (0.2 mol) in ethanol (20 mL) was added hydroxylamine hydrochloride (0.4 mol) in water (40 mL). Then anhydrous sodium carbonate(0.4 mol) in water (120 mL) was slowly added to the resulting solution and the mixture was stirred at 358k for 5 h and then concentrated under vacuum to evaporate some water. The resulting suspension was filtered, the solid that formed was washed with cold water and dried under vacuum. Block-shaped crystals suitable for X-ray diffraction were obtained from methanol.

### Refinement

H atoms bound to N and O were located in difference Fourier maps and refined isotropically with *U*_iso_(H) = 1.2*U*_eq_(N) [1.5*U*_eq_(O)]. H atoms attached to C were added at their calculated positions and included in the structure factor calculations, with C—H = 0.93Å (aromatic) and 0.97 Å (methylene), and with *U*_iso_(H) = 1.2*U*_eq_(C). The F atoms of the CF_3_ group were disordered over two positions. Occupancy was fixed at 0.5 for each component in the final refinement cycles.

### Crystal data

**Table:**

C_8_H_7_F_3_N_2_O	*F*(000) = 416
*M**_r_* = 204.16	*D*_x_ = 1.501 Mg m^−^^3^
Monoclinic, *P*2_1_/*c*	Mo *K*α radiation, λ = 0.71073 Å
Hall symbol: -P 2ybc	Cell parameters from 4741 reflections
*a* = 9.8706 (8) Å	θ = 3.1–27.4°
*b* = 11.2540 (12) Å	µ = 0.14 mm^−^^1^
*c* = 8.4033 (7) Å	*T* = 293 K
β = 104.61 (2)°	Irregular block, colorless
*V* = 903.29 (16) Å^3^	0.32 × 0.24 × 0.20 mm
*Z* = 4	

### Data collection

**Table:**

Rigaku R-AXIS RAPID diffractometer	2058 independent reflections
Radiation source: fine-focus sealed tube	1324 reflections with *I* > 2σ(*I*)
graphite	*R*_int_ = 0.077
Detector resolution: 10.0 pixels mm^-1^	θ_max_ = 27.4°, θ_min_ = 3.1°
ω scans	*h* = −12→12
Absorption correction: multi-scan (*ABSCOR*; Higashi, 1995)	*k* = −14→14
*T*_min_ = 0.946, *T*_max_ = 0.972	*l* = −10→10
8605 measured reflections	

### Refinement

**Table:**

Refinement on *F*^2^	Primary atom site location: structure-invariant direct methods
Least-squares matrix: full	Secondary atom site location: difference Fourier map
*R*[*F*^2^ > 2σ(*F*^2^)] = 0.056	Hydrogen site location: inferred from neighbouring sites
*wR*(*F*^2^) = 0.183	H atoms treated by a mixture of independent and constrained refinement
*S* = 1.07	*w* = 1/[σ^2^(*F*_o_^2^) + (0.101*P*)^2^ + 0.058*P*] where *P* = (*F*_o_^2^ + 2*F*_c_^2^)/3
2058 reflections	(Δ/σ)_max_ < 0.001
164 parameters	Δρ_max_ = 0.23 e Å^−^^3^
42 restraints	Δρ_min_ = −0.29 e Å^−^^3^

### Special details

**Table:**

Geometry. All e.s.d.'s (except the e.s.d. in the dihedral angle between two l.s. planes) are estimated using the full covariance matrix. The cell e.s.d.'s are taken into account individually in the estimation of e.s.d.'s in distances, angles and torsion angles; correlations between e.s.d.'s in cell parameters are only used when they are defined by crystal symmetry. An approximate (isotropic) treatment of cell e.s.d.'s is used for estimating e.s.d.'s involving l.s. planes.
Refinement. Refinement of *F*^2^ against ALL reflections. The weighted *R*-factor *wR* and goodness of fit *S* are based on *F*^2^, conventional *R*-factors *R* are based on *F*, with *F* set to zero for negative *F*^2^. The threshold expression of *F*^2^ > σ(*F*^2^) is used only for calculating *R*-factors(gt) *etc*. and is not relevant to the choice of reflections for refinement. *R*-factors based on *F*^2^ are statistically about twice as large as those based on *F*, and *R*- factors based on ALL data will be even larger.

### Fractional atomic coordinates and isotropic or equivalent isotropic displacement parameters (Å^2^)

**Table:**

	*x*	*y*	*z*	*U*_iso_*/*U*_eq_	Occ. (<1)
C1	−0.0030 (2)	0.6553 (2)	0.2394 (3)	0.0759 (7)	
F1A	−0.0355 (6)	0.7584 (3)	0.1633 (6)	0.1154 (16)	0.783 (15)
F2A	−0.0071 (5)	0.5782 (6)	0.1205 (6)	0.1246 (19)	0.783 (15)
F3A	−0.1063 (3)	0.6322 (8)	0.3082 (5)	0.1140 (18)	0.783 (15)
F2B	−0.082 (2)	0.564 (2)	0.260 (3)	0.133 (8)	0.217 (15)
F3B	−0.0726 (17)	0.7557 (13)	0.244 (4)	0.133 (8)	0.217 (15)
F1B	−0.0132 (15)	0.629 (2)	0.0833 (8)	0.119 (6)	0.217 (15)
C2	0.1369 (2)	0.65458 (19)	0.3615 (3)	0.0581 (6)	
C3	0.2008 (2)	0.7593 (2)	0.4227 (3)	0.0620 (6)	
H3	0.1607	0.8316	0.3828	0.074*	
C4	0.3252 (2)	0.75674 (18)	0.5442 (3)	0.0575 (6)	
H4	0.3685	0.8276	0.5857	0.069*	
C5	0.3858 (2)	0.64908 (16)	0.6046 (2)	0.0484 (5)	
C8	0.5128 (2)	0.64594 (16)	0.7435 (2)	0.0503 (5)	
N1	0.6164 (2)	0.72612 (19)	0.7499 (3)	0.0731 (6)	
H1N	0.620 (3)	0.764 (3)	0.665 (4)	0.088*	
H2N	0.689 (3)	0.717 (2)	0.830 (4)	0.088*	
N2	0.51210 (16)	0.56917 (14)	0.85708 (19)	0.0515 (5)	
O1	0.63766 (15)	0.57836 (13)	0.98654 (18)	0.0622 (5)	
H1O	0.618 (3)	0.532 (2)	1.059 (3)	0.093*	
C6	0.3222 (2)	0.54448 (19)	0.5390 (3)	0.0622 (6)	
H6	0.3633	0.4720	0.5765	0.075*	
C7	0.1980 (2)	0.5470 (2)	0.4181 (3)	0.0679 (7)	
H7	0.1554	0.4763	0.3747	0.081*	

### Atomic displacement parameters (Å^2^)

**Table:**

	*U*^11^	*U*^22^	*U*^33^	*U*^12^	*U*^13^	*U*^23^
C1	0.0610 (14)	0.104 (2)	0.0610 (14)	0.0069 (14)	0.0127 (11)	0.0105 (14)
F1A	0.099 (2)	0.128 (3)	0.101 (3)	0.0203 (16)	−0.0095 (18)	0.0460 (19)
F2A	0.105 (2)	0.156 (4)	0.085 (2)	0.026 (2)	−0.027 (2)	−0.034 (2)
F3A	0.0503 (12)	0.200 (5)	0.0912 (19)	0.0029 (18)	0.0178 (11)	0.032 (2)
F2B	0.071 (8)	0.173 (13)	0.131 (12)	−0.053 (8)	−0.019 (7)	0.066 (9)
F3B	0.075 (7)	0.144 (11)	0.154 (15)	0.039 (7)	−0.020 (8)	−0.008 (9)
F1B	0.061 (5)	0.216 (15)	0.075 (6)	0.026 (7)	0.010 (4)	0.049 (8)
C2	0.0521 (11)	0.0711 (14)	0.0523 (11)	0.0048 (10)	0.0152 (9)	0.0074 (10)
C3	0.0615 (13)	0.0589 (13)	0.0664 (13)	0.0101 (10)	0.0177 (11)	0.0179 (10)
C4	0.0622 (12)	0.0453 (11)	0.0645 (12)	−0.0022 (9)	0.0149 (10)	0.0077 (9)
C5	0.0508 (11)	0.0461 (11)	0.0500 (10)	0.0000 (8)	0.0161 (8)	0.0033 (8)
C8	0.0513 (11)	0.0430 (10)	0.0567 (11)	0.0002 (8)	0.0136 (9)	−0.0008 (8)
N1	0.0646 (12)	0.0725 (13)	0.0765 (14)	−0.0214 (10)	0.0071 (10)	0.0161 (11)
N2	0.0519 (9)	0.0474 (9)	0.0516 (9)	0.0000 (7)	0.0062 (7)	0.0021 (7)
O1	0.0586 (9)	0.0609 (10)	0.0585 (9)	−0.0033 (7)	−0.0009 (7)	0.0033 (7)
C6	0.0634 (13)	0.0451 (11)	0.0709 (14)	0.0038 (9)	0.0035 (11)	0.0016 (9)
C7	0.0663 (14)	0.0583 (13)	0.0718 (14)	−0.0050 (10)	0.0042 (11)	−0.0059 (10)

### Geometric parameters (Å, °)

**Table:**

C1—F2A	1.316 (3)	C4—H4	0.9300
C1—F3A	1.318 (3)	C5—C6	1.381 (3)
C1—F1B	1.323 (3)	C5—C8	1.483 (3)
C1—F1A	1.324 (3)	C8—N2	1.289 (2)
C1—F3B	1.328 (3)	C8—N1	1.354 (3)
C1—F2B	1.330 (3)	N1—H1N	0.84 (3)
C1—C2	1.498 (3)	N1—H2N	0.86 (3)
C2—C3	1.374 (3)	N2—O1	1.432 (2)
C2—C7	1.383 (3)	O1—H1O	0.86 (3)
C3—C4	1.385 (3)	C6—C7	1.382 (3)
C3—H3	0.9300	C6—H6	0.9300
C4—C5	1.389 (3)	C7—H7	0.9300
			
F2A—C1—F3A	108.9 (3)	C2—C3—C4	119.77 (18)
F2A—C1—F1B	28.5 (8)	C2—C3—H3	120.1
F3A—C1—F1B	121.1 (7)	C4—C3—H3	120.1
F2A—C1—F1A	104.7 (3)	C3—C4—C5	120.46 (18)
F3A—C1—F1A	105.3 (3)	C3—C4—H4	119.8
F1B—C1—F1A	76.5 (9)	C5—C4—H4	119.8
F2A—C1—F3B	131.8 (9)	C6—C5—C4	119.17 (19)
F3A—C1—F3B	72.1 (12)	C6—C5—C8	120.16 (17)
F1B—C1—F3B	107.9 (10)	C4—C5—C8	120.59 (17)
F1A—C1—F3B	37.4 (13)	N2—C8—N1	124.23 (19)
F2A—C1—F2B	71.5 (14)	N2—C8—C5	115.93 (16)
F3A—C1—F2B	41.1 (14)	N1—C8—C5	119.69 (18)
F1B—C1—F2B	93.3 (11)	C8—N1—H1N	120 (2)
F1A—C1—F2B	131.5 (9)	C8—N1—H2N	115.4 (18)
F3B—C1—F2B	109.3 (11)	H1N—N1—H2N	121 (3)
F2A—C1—C2	111.3 (3)	C8—N2—O1	110.41 (15)
F3A—C1—C2	112.3 (2)	N2—O1—H1O	100.9 (19)
F1B—C1—C2	120.3 (6)	C5—C6—C7	120.37 (19)
F1A—C1—C2	113.9 (2)	C5—C6—H6	119.8
F3B—C1—C2	112.2 (7)	C7—C6—H6	119.8
F2B—C1—C2	112.1 (6)	C6—C7—C2	120.0 (2)
C3—C2—C7	120.2 (2)	C6—C7—H7	120.0
C3—C2—C1	120.58 (19)	C2—C7—H7	120.0
C7—C2—C1	119.1 (2)		
			
F2A—C1—C2—C3	137.2 (4)	C2—C3—C4—C5	0.0 (3)
F3A—C1—C2—C3	−100.5 (5)	C3—C4—C5—C6	1.6 (3)
F1B—C1—C2—C3	107.0 (12)	C3—C4—C5—C8	−175.20 (18)
F1A—C1—C2—C3	19.2 (4)	C6—C5—C8—N2	−41.5 (3)
F3B—C1—C2—C3	−21.5 (17)	C4—C5—C8—N2	135.3 (2)
F2B—C1—C2—C3	−145.0 (19)	C6—C5—C8—N1	142.6 (2)
F2A—C1—C2—C7	−46.0 (5)	C4—C5—C8—N1	−40.6 (3)
F3A—C1—C2—C7	76.4 (5)	N1—C8—N2—O1	−2.0 (3)
F1B—C1—C2—C7	−76.1 (12)	C5—C8—N2—O1	−177.71 (15)
F1A—C1—C2—C7	−164.0 (4)	C4—C5—C6—C7	−1.7 (3)
F3B—C1—C2—C7	155.3 (17)	C8—C5—C6—C7	175.06 (19)
F2B—C1—C2—C7	31.9 (19)	C5—C6—C7—C2	0.3 (4)
C7—C2—C3—C4	−1.5 (3)	C3—C2—C7—C6	1.3 (4)
C1—C2—C3—C4	175.34 (19)	C1—C2—C7—C6	−175.5 (2)

### Hydrogen-bond geometry (Å, °)

**Table:**

*D*—H···*A*	*D*—H	H···*A*	*D*···*A*	*D*—H···*A*
N1—H1N···O1^i^	0.84 (3)	2.36 (3)	3.165 (3)	161 (3)
O1—H1O···N2^ii^	0.86 (3)	1.98 (3)	2.766 (2)	152 (3)

Symmetry codes: (i) *x*, −*y*+3/2, *z*−1/2; (ii) −*x*+1, −*y*+1, −*z*+2.
